# Biophysical characteristics and thermophysiological responses at the inflection point in deep body temperature for individuals with high or low aerobic fitness

**DOI:** 10.1186/2046-7648-4-S1-A51

**Published:** 2015-09-14

**Authors:** Ella F Walker, Jo Corbett, James R House, Michael J Tipton

**Affiliations:** 1 Extreme Environments Laboratory, Department of Sport and Exercise Science, University of Portsmouth, Portsmouth, UK

## Introduction

Endurance training increases aerobic fitness, improves thermoregulatory function [1] and exercise tolerance in the heat [2]. However, studies comparing individuals of high and low aerobic fitness during exercise in the heat are often confounded by differences in body weight and body composition [3]. It is also disputed if using the relative work rate to standardise exercise intensity is appropriate [4,5] as individuals with higher aerobic fitness will be working at a higher absolute work rate with increased heat production. This study examined the influence of aerobic fitness on biophysical characteristics and thermophysiological responses when moving from compensable to uncompensable conditions, at matched relative and absolute work rates, under conditions of high and low humidity.

## Methods

Eight high (HI) (VO_2max _= 58.73[6.28] mL.kg^-1^.min^-1^) and eight low aerobic fitness (LO) (46.53[6.73] mL.kg^-1^.min^-1^) males, matched for body mass, body surface area and % body fat volunteered. LO exercised at 60 W (ABS) in a hot-humid (28 °C starting temperature, 80 % rh [HH]) and hot-dry (34 °C starting temperature, 20 % rh [HD]) environment. HI completed the same trials, plus an additional trial in each condition to match the relative intensity of the LO group exercising at 60 W (REL). T_db _was incremented after 60 minutes of exercise until an inflection in rectal temperature (T_reinfl_). T_db_, T_re_, upper back sweat rate (SR_Back_) and forearm skin blood flow (SkBF) were measured at the T_reinfl_. Data were analysed by mixed-model ANOVA, with *post hoc *analysis by paired (effect of humidity) or independent t-tests (effect of fitness).

## Results

T_db _at T_re _inflection was lower in the HH condition in both ABS (p < 0.001) and REL (p < 0.001) conditions. Additionally, during REL trials, T_db _at T_reinfl _was lower for HI than LO in the HH condition (p = 0.013). T_re _was lower in HI than LO at the T_reinfl _in the HH condition during ABS trials (p = 0.010), with no effect of fitness or humidity condition in REL trials. HI had higher SR_Back _at the T*_r_*_einfl _in in both ABS trials (HH, p = 0.030; HD, p = 0.005) and REL trials (HH, p = 0.001; HD condition, p < 0.001). SkBF at the T_reinfl _not differ between any of the trials (p > 0.05).

## Discussion

There was no effect of fitness on T_db _at T_reinfl _at ABS, but HI had a lower T_db _at T_reinfl _at REL in the HH condition reflecting the higher metabolic heat production and lower capacity for evaporative heat loss. The difference in T_re _at the inflection point between HI and LO, in the HH environment, may be due to the need to maintain a gradient for heat exchange from core to shell under conditions where an increased SR is not sufficient to maintain heat loss. Nonetheless, HI individuals have been shown to tolerate higher T_c _temperatures than LO counterparts when exercising in the heat [2] and the practical significance of the lower T_re _at the inflection point is unclear.

## Conclusion

Despite improved thermoregulatory function, when body weight and body composition are controlled, aerobic fitness does not offer any benefit in terms of the biophysical conditions eliciting the transition to uncompensable heat stress when exercising a given ABS. Moreover, the T_reinfl _may occur at a lower T_db _in HI individuals when working at matched REL under conditions that limit evaporative heat loss. Finally, HI individuals may show a T_reinfl _at a lower T_re _during exercise at a given ABS in HH conditions.
